# Smartphone-Based Remote Self-Management of Chronic Low Back Pain: A Preliminary Study

**DOI:** 10.1155/2019/4632946

**Published:** 2019-02-06

**Authors:** Jingyi Yang, Quan Wei, Yanlei Ge, Lijiao Meng, Meidan Zhao

**Affiliations:** ^1^The Department of Rehabilitation Medicine, West China Hospital, Sichuan University, Chengdu, China; ^2^College of Acupuncture and Massage, Tianjin University of Traditional Chinese Medicine, Tianjin, China

## Abstract

**Objective:**

To assess the additional effect of self-management on physiotherapy via the use of APPS on management of chronic low back pain.

**Method:**

A single-blinded randomized control trial was conducted. 8 participants (male: 4; female: 4) were recruited from the Rehabilitation Clinic of The Hong Kong Polytechnic University. Participants in the treatment group received self-management plus physiotherapy and the control group received physiotherapy only. Assessment was carried out pretreatment, midterm (week 2), and posttreatment (week 4), including Visual Analog Scale (VAS), Pain Self-Efficacy Questionnaire (PSEQ), Roland Morris Disability Questionnaire (RMDQ), and SF36.

**Results:**

Compared with the physiotherapy group, the self-management plus physiotherapy group had significance in PSEQ (*p*=0.035), RMDQ (*p*=0.035), SF36-Bodily Pain (*p*=0.008), and SF36-Mental Health (*p*=0.013). VAS showed a positive trend although there was no significant difference.

**Conclusion:**

This pilot study indicated that smartphone APPS-based self-management program appears to bring additional benefits to physiotherapy for patients with CLBP. Self-management is a potential approach for people with CLBP.

## 1. Background of Research

Low back pain (LBP) is a common condition with the lifetime prevalence around 60% to 85% in industrialized societies [1–3], about 5% of them will develop chronic low back pain (CLBP) [4, 5]. Low back pain is also one of the most frequent symptoms complained by patients in Hong Kong [6]. LBP can increase healthcare costs and working and functional disability as well as leading to psychological disorders [1, 7–9]. These may result in the decline of physical function and work ability which are likely to increase the burdens on the patients themselves, their families, and the society [10]. Therefore, LBP has become the public health concern nowadays.

Management approaches for LBP vary from surgery, medication, complementary and alternative therapy, physiotherapy, and self-management [11]. In 2017, the American College of Physicians provided a noninvasive treatment guideline for low back pain which is the preferred treatment [12]. In recent years, an increasing attention has been paid to the self-management that focuses on the active participation of the patients instead of traditional strategies that mainly involves passive recipient of intervention [13–16]. LBP is easy to recur, so patients tend to rely on health professionals for offering help, which may be effective in controlling pain for a short period time but is more likely to initiate dependent trend [13, 16]. Therefore, self-management, as a continuous process with cost-effective and patient-focused strategy, has complemented traditional models of care to sustain well-being for patients in their foreground perspective [17, 18].

“Self-management refers to the individual's ability to manage symptoms, treatment, physical and psychosocial consequences, and lifestyle changes inherent in living with a chronic condition and to affect the cognitive, behavioral, and emotional responses necessary to maintain a satisfactory quality of life. Thus, a dynamic and continuous process of self-regulation is established” [19, 20]. The definition of self-management has been divided into two aspects: firstly, it adopts a thorough autonomic self-care model. Secondly, it involves primarily self-care; at the same time, clinicians usually communicate with patients and give them appropriate advice and support [17]. It is viewed as a healthcare model that promotes patients to learn skills and monitor their health condition and self-efficacy in daily life as well as obtain a primary role in the self-management [21]. It mainly contains the following skills: problem solving, decision making, resource finding and utilizing, partnership forming between the patient and the professionals, self-tailoring, and action taking. Also, three tasks are involved, including medical or behavioral management, role management, and emotional management [14, 22]. The core of self-management program for chronic LBP should be exercise, with the support of health education and professional advice. A recent systematic review concluded that exercise is an effective treatment to manage LBP, both short-term and long-term effects [17]. In addition, Coon et al. found the best self-management model should take physical activity behavior as the aim, which refers to self-monitoring, goal setting, feedback, and the consequence [23].

With its advancement, information technologies assist the implementation of self-management for chronic condition. Specifically, the literatures have proved that self-management plays an essential role in managing chronic conditions, such as diabetes, hypertension, asthma, cardiovascular disease, hemophilia, and so forth, via e-mail, electronic diaries, website, telephone coaching, and software of smartphone to transmit and record the health information, especially in cooperation with physicians [15, 24–33]. Among them, the most popular applications are web based and APPS based. A few studies have shown that the web-based management has shown to be effective [15, 30, 33]. A systematic review showed that self-management program is a good choice for patients with CLBP to manage their symptom, due to its safety as well as effectiveness in pain intensity and disability [34]. Compared with traditional pattern, internet-related approach is more convenient. Therefore, this study designed a self-management program for CLBP that reminds the participants to perform exercise and collected their feedback on pain intensity and activity level through an APP on smartphone. This may increase the awareness of our subjects on postural correction and remind them to perform exercises, which are important elements in self-management for low back pain.

Therefore, the objective of this study is to assess the additional effect of self-management on physiotherapy through the application of APPS on management of chronic low back pain.

## 2. Methodology

### 2.1. Subjects

A single-blinded randomized controlled trial was conducted. The randomization was conducted by drawing nonreplacement lots from an envelope, and subjects were randomly allocated to either the self-management group (SM + PT) or physiotherapy group (PT). All subjects received 4-week physiotherapy, while the SM + PT group received self-management program through the use of an APP. Subjects in the control group received physiotherapy only. Participants in this study were recruited from the Rehabilitation Clinic of the Hong Kong Polytechnic University.

Subjects aged from 18 or above with confirmed diagnosis of chronic low back pain (>3 months) by physicians were recruited. Inclusion criteria were people with nonspecific low back pain due to musculoskeletal origins. Also, the subject should have a smart mobile phone operated by the Android system or Apple store that allows the download of the APPS, and they should be able to perform a brief exercise during regular working hour. Exclusion criteria included history of receiving major surgery. All subjects provided written informed consent prior to the study.

### 2.2. Treatment Protocol

The project was approved by the research ethical committee with the reference number: 880_RO. The subjects were randomly allocated into the self-management group (SM + PT) or physiotherapy (PT) group. The treatment group received the physiotherapy plus the self-management program during the study period, and the control group received PT only. The PT may consist of manual therapy, electrophysical therapy, and traction as prescribed by the physiotherapist.

The core component of our self-management program was exercises. Individualized exercise was prescribed to each subject by their own therapist; the therapist may modify the exercises according to subjects' feedback and symptom over time. Subjects in the SM + PT group were reminded to perform exercises 4 times daily for 4 weeks, i.e., when the subject got up in the morning, morning break at work, afternoon break, and before going to bed. Personalized time slots designated for performing exercise were worked out for subjects based on their own work pattern and personal schedule. A reminder of exercise and pain diary will be sent to each subject via an APP called Pain Care which can be downloaded for free from the app store for both the Android and Apple system. This APP contains mainly three elements. In the “New Pain Episode,” the subject can choose the date and input the pain intensity and activity levels, and the subject can put down remark before and after each exercise session. In the Personal Report, the subject can retrieve their own data that had been inputted earlier. The subjects can access online at http://my.ringful.com and select “view data” to view and print out their data and share data with the assessor by sending an e-mail to both the assessor and the user (subject). In the “About & Tools,” people can tailor make the setting of the reminders, including none, every hour, every 2 hours, every 4 hours, every 8 hours, or every day, or just set alarm via the mobile phone. Active participation of patients in the rehabilitation process is essential for the self-management program. This can increase patients' motivation in performing exercise, which can bring benefits to managing chronic low back pain.

Assessment was conducted pretreatment, midterm (week 2), and posttreatment (week 4), including the use of Visual Analog Scale (VAS), Pain Self-Efficacy Questionnaire (PSEQ), Roland Morris Questionnaire (RMDQ), and Short Form Health Survey (SF36).

### 2.3. Outcome Measures

All assessments were done at the baseline, midterm, and end of week 4.

1. Present pain intensity was registered by Visual Analog Scale (VAS), which presented with a horizontal line of 100 mm in length, anchored by word descriptors at each end. The subjects made a mark on the horizontal line that represented their perception of pain at that moment. The VAS score was determined by measuring the distance from the left hand end of the line to the point that the patient marks. VAS has been proven to be a valid and reliable instrument for registering pain intensity [35–37].
2. The self-efficacy of patients was measured by Pain Self-Efficacy Questionnaire (PSEQ), which contains 10 items; each item scores ranged from 0 (no confidence) to 7 (complete confidence). A higher score represents better self-efficacy during their lives. The validity and reliability of PSEQ have been validated for the assessment of chronic pain [38].
3. Disability level was assessed by Roland Morris Disability Questionnaire (RMDQ), which is widely utilized in the clinical setting to evaluate low back pain. This instrument has been shown to be valid and reliable for assessing health status. It consists of 24 items that require the subjects to report the effects of the low back pain on their daily lives and function. Each question is marked by “0 (disagree with the item)” or “1 (agree the item).” The total score ranged from 0 (normal) to 24 (dysfunction) [39–42]. The questionnaire was finished by the patients and scored by the assessor.
4. The health-related quality of life was assessed via SF36 that consists of 36 items and could be divided to nine aspects, namely physical functioning, role physical, bodily pain, general health, vitality, social functioning, role emotional, mental health, and health transition. The score was calculated and analyzed according to the sorts it belongs to, and a higher score indicates better physical condition. It has been demonstrated to be valid and reliable and can benefit a wide range of patients [6, 43, 44].

### 2.4. Data Analyses

The data were analyzed by using the Statistical Package for Social Science (SPSS) [45]. Normality was checked before data analysis. Two-way repeated measures ANOVA were conducted to examine the effect of self-management on CLBP, and ANCOVA was performed if covariant was found at the baseline. If the dataset did not show normal distribution, the Friedman test was used to examine the difference between groups over time. Post hoc test for ANCOVA was Bonferroni, and signed-ranks test was for Friedman test. The intention-to-treat approach was applied to handle the missing data. Level of each statistical test was set at *α* = 0.05.

## 3. Results

### 3.1. Participants

At the beginning of the project, 12 subjects were included in the study, and 4 of them failed to join in due to personal reason. Finally, 8 subjects participated in the present study with 5 in the SM + PT group and 3 in the PT group. In the SM + PT group, 3 participants completed all assessment, and 2 of them dropped out after finishing the midterm assessment. In the PT group, all subjects completed all assessment. Intent-to-treat analysis was performed for subsequent data analysis.

The baseline data of the subjects are presented in Table 1. The mean age of the sample was 35 years old (SD = 10.93) and 50.33 years old (SD = 9.29), and the mean of pain duration was 35.80 months (SD = 54.39) and 17 months (SD = 17.06) in the SM + PT group and PT group, respectively. The demographic data were comparable between the two groups. The results of the mean (standard deviation (SD)) are presented in Table 2. There was no significant difference between the two groups in terms of VAS, PSEQ, RMDQ, and SF36 except SF36-Vitality (SF36-VT) (*p*=0.036).

### 3.2. Treatment Effects

#### 3.2.1. Visual Analog Scale (VAS)

There was no significant difference in group effects (*p*=0.24) and within-group effects (see Table 3). However, our findings suggest that self-management seems to provide additional benefits to PT for people with CLBP. The VAS of the SM + PT group decreased from 5.0 to 3.4, but the VAS of the PT group remained around 6 from the baseline to posttreatment.

#### 3.2.2. Pain Self-Efficacy Questionnaire (PSEQ)

At the beginning, PSEQ score for group effects showed significant difference (*p*=0.035) but no difference in interaction contrast (*p*=0.076) and within-group effects. After putting SF36-VT as covariant, the adjusted PSEQ did not indicate significant difference for group effects (*p*=0.18), but the interaction contrast (0.008) and mid-post within-group effects (*p*=0.033) showed significance in the adjusted PSEQ (Table 3). Nevertheless, the PSEQ score in the SM + PT group showed an increasing trend (mean: pretreatment 38.6; midterm 46.8; posttreatment 47.6) while the PT group had a decreasing trend (mean: pretreatment 34.3; midterm 33.3; posttreatment 30.7).

#### 3.2.3. Roland Morris Disability Questionnaire (RMDQ)

At first, the RMDQ score showed a significant difference between the groups (*p*=0.035). By entering SF36-VT as covariant, the adjusted RMDQ indicated no significant difference between two groups (*p*=0.16). Both RMDQ and adjusted RMDQ showed no statistically significant in terms of within-group effects and within-group contrast (Table 3). However, subjects in the SM + PT group revealed a positive response to the self-management by saying “feel good” or “better” after doing exercises.

#### 3.2.4. SF36

The Friedman test was conducted to test the SF36-Physical Function (SF36-PF), SF36-Role Physical (SF36-RP), SF36-Bodily Pain (SF36-BP), SF36-Vitality (SF36-VT), SF36-Social Function (SF36-SF), SF36-Role Emotional (SF36-RE), and SF36-Mental Health (SF36-MH); two-way repeated ANCOVA was used to evaluate SF36-General Health (GH) because this was the only item that showed normal distribution in SF36.

Output of Friedman test demonstrated overall significant difference with *p* value = 0.008 across the three time occasions in SF36-BP. By a post hoc test, significant difference was detected in SF36-BP between two time intervals in the pretreatment and midterm (*p*=0.023) as well as pretreatment and posttreatment (*p*=0.046), but no significant difference was found in midterm and posttreatment (*p*=0.102). There was no significant difference between groups at the baseline, midterm, or posttreatment (all *p* > 0.05).

Based on the analysis, SF36-MH revealed an overall significant improvement with *p*=0.013. Then, the post hoc test showed an overall significance between the two groups in comparison of the baseline and posttreatment (*p*=0.017). Difference between group indicated no statistical difference between two groups in the pretreatment, midterm, and posttreatment, respectively (*p* > 0.05). SM + PT group indicated a significant within-group difference between the pretreatment and midterm (*p*=0.046).

For the adjusted SF36-GH, pretreatment and posttreatment within-group effects were detected to be significant difference (*p*=0.033).

However, no significant group effects were found in the other six subscales (SF36-PF, *p*=0.87; SF36-RP, *p*=0.48; SF36-GH, *p*=0.124; adjusted SF36-GH, *p*=0.88; SF36-VT, *p*=0.55; SF36-SF, *p*=0.82; SF36-RE, *p*=0.16), and within-group effects also showed no significant difference in these items (Table 4) except the pre-post effects on the adjusted SF36-GH.

## 4. Discussion

In this randomized controlled trial, APP integrating self-management was commendable to physiotherapy alone for improvement in CLBP. In the SM + PT group, bodily pain and mental health revealed significant improvement over time, and interaction effect of the adjusted PSEQ improved significantly. Meanwhile, the VAS, PSEQ, and RMDQ also indicated an increasing trend across the treatment session. However, the group difference did not reach significance. Since the PT group also received physiotherapy that may reduce pain and disability, both groups showed improvement over time. This may explain why there was no significant difference between groups.

Improvement of VAS was feasible compared to other outcome measurements on CLBP patients [15, 46]. In the study of Schulz et al., an internet-based self-management was designed to detect the effectiveness of CLBP for five months, and no significant difference was found in both groups. Previous studies suggested that self-management program could bring benefit to physical and psychological function but did not reach statistical significance in pain reduction [47–49]. In the study of Schulz, participants reported a decrease in pain intensity measured by VAS but not for the control group. In our study, the average VAS ranged from 5 to 3.4 in the SM + PT group, while remained around 6 in the PT group. This implies that even though the group difference did not reach a significant level, the self-management program plays a positive role in reducing pain intensity.

Iles and her colleagues conducted a study and provided telephone coach-based approach for patients with nonspecific low back pain [32]; they assessed PSEQ at the baseline, week 4, and week 12. In their study, the treatment effect showed a linear trend; the self-efficacy of the treatment group improved during the study period; however, it did not reach statistical significance. Nevertheless, in our study, PSEQ showed significant difference in group effects (*p*=0.035), and the adjusted PSEQ showed midterm and posttreatment group effects and interaction effects but not significant in group effects (*p*=0.180). There may be three potential reasons for the explanation. First, in the experiment of Iles, telephone coaching was applied once per day for a total of 4 weeks; the frequency is much lower than that in our project, which reminded the participants 4 times per day for a total of 4 weeks. It is possible that more frequent reminder sent to the subjects would produce greater improvement. Second, Iles and her colleagues provided coaching to subjects that mainly provided health advice and some motivational or cognitive information; they did not contain any intervention suggestion. In contrast, the SM + PT group of our study received tailored exercises. It appears that exercises are more effective intervention than the psychological advice alone. Third, it seems that it is more convenient and attractive for patients to receive reminder via an APP than telephone call, so it is not surprising that patients in our SM + PT group have a relatively better result than did the previous study conducted by Iles. In our study, the PT group showed a decreasing trend after midterm assessment, and the potential reason may be they discontinued the treatment in the clinic. Further studies about the use of APPS should be performed to observe the effectiveness of this high-tech strategy on managing clinical pain conditions. But generally, self-management is a promising strategy for people with low back pain regardless of telephone coaching or APPS.

Disability level assessed by RMDQ in our study was a little bit different from what Von et al. [46] have done in an earlier study. RMDQ score in their study was significantly different in six months and twelve months [46]. So, duration may be a potential reason for the explanation based on the findings. In further study, a longer duration study should be conducted to evaluate the changes of RMDQ.

There is limited evidence to contrast the improvements of self-management on various subscales of SF36. Frost and her colleagues found that participants showed more promotions in mental health and physical functioning at two months in treatment group than the control group, but not statistically significant [50]. In contrast, our study reported a statistically significant advantage for SF36-BP and SF36-MH in terms of group effects and the adjusted pretreatment and posttreatment effects on SF36-GH. We cannot identify the reason for different results, but it is possible that the research approach may contribute to the impact. Specifically, in a study conducted by Frost, patients in the treatment group received physiotherapy plus counseling, and participants in the control group were treated by counseling alone. On one hand, patients are more likely to benefit from PT + SM, especially when the self-management consists of individualized exercises than the physiotherapy + counseling. On the other hand, individuals tend to obtain more psychological effects after being treatment by relatively comprehensive strategy, since people are required to give a feedback about their pain level and activity level after each exercise; this may remind them that they are being treated by self-management.

However, the self-management program has attracted more and more attention in recent years. Because it helps to save time burden as well as economic costs of hospitalization for this population. Alicia compared interactive voice response-based self-management with cognitive behavioral therapy for patients with CLBP. The outcome showed both strategies improved in pain intensity and physical condition. Whereas taking into account advantages of self-management program, it can be considered to replace traditional methods [51].

There are several limitations in this study. First, a small sample size may result in a lower credibility. Second, short treatment duration of data collection may affect the outcomes. Third, no follow-up was included, so it is difficult to compare the long-term effects of self-management. Finally, we only included people who have smartphone, who may have higher education and social status. Future study with a larger sample size and longer study period is necessary.

As a conclusion, our study demonstrated that smartphone APPS-based self-management program appears to offer additional benefits to physiotherapy for patients with CLBP. More powerful studies should be conducted in the future to assess the effects of self-management on CLBP.

## Figures and Tables

**Table 1:** Baseline of subjects' characteristics.

Variable	Mean (SD)		*p*
	SM + PT (*N*=5)	PT (*N*=3)	
Age	35.00 (10.93)	50.33 (9.29)	0.090
Gender	Male = 4, Female = 1	Female = 3	0.071
Height (cm)	172.80 (7.40)	162.67 (6.43)	0.098
Weight (kg)	64.80 (10.31)	62.00 (15.88)	0.77
Pain duration (month)	35.80 (54.39)	17.00 (17.06)	0.79
Activity level	1.80 (0.45)	1.33 (0.58)	0.39
VAS	5.00 (1.87)	6.00 (1.00)	0.43
PSEQ	38.60 (8.50)	34.33 (8.02)	0.51
RMDQ	6.00 (3.74)	12.00 (3.61)	0.068

SF36			
Physical function	74.00 (21.62)	46.67 (28.87)	0.25
Role physical	20.00 (20.92)	16.67 (14.43)	1.00
Bodily pain	44.00 (18.17)	63.33 (5.77)	0.14
General health	49.00 (11.40)	58.33 (20.21)	0.43
Vitality	50.00 (0.00)	63.33 (5.77)	**0.036** ^*∗*^
Social function	52.50 (10.16)	45.83 (7.22)	0.39
Role emotional	39.87 (36.55)	22.22 (38.49)	0.57
Mental health	58.40 (13.15)	66.67 (2.31)	0.39

VAS: Visual Analog Scale; PSEQ: Pain Self-Efficacy Questionnaire; RMDQ: Roland Morris Disability Questionnaire; SF36: Short Form Health Survey; ^*∗*^*p* < 0.05.

**Table 2:** Comparisons of various outcomes between groups over time.

Outcome measure	Mean (SD)											
	Pretreatment				Midterm				Posttreatment			
	SM + PT		PT		SM + PT		PT		SM + PT		PT	
VAS	5.00	1.87	6.00	1.00	4.00	2.55	6.67	0.58	3.40	2.88	6.00	1.73
PSEQ	38.60	8.50	34.30	8.02	46.80	6.26	33.30	3.79	47.60	7.13	30.70	5.51
RMDQ	6.00	3.74	12.00	3.61	5.20	2.78	12.30	4.16	4.40	3.05	11.70	5.69

SF36												
Physical function	74.00	21.62	46.67	28.87	80.00	13.69	51.67	15.28	59.00	61.89	51.67	18.93
Role physical	20.00	20.92	16.67	14.43	45.00	37.08	8.33	14.43	45.00	37.08	25.00	43.30
Bodily pain	44.00	18.17	63.33	5.77	34.00	15.17	53.33	5.77	40.00	14.14	56.67	5.77
General health	49.00	11.40	58.33	20.21	48.00	10.37	61.67	12.58	50.00	7.91	65.00	5.00
Vitality	50.00	0.00	63.33	5.77	51.00	14.75	71.67	10.41	47.00	12.55	65.00	5.00
Social function	52.50	10.16	45.83	7.22	50.00	12.25	50.17	12.25	52.40	10.34	50.17	12.25
Role emotional	39.87	36.55	22.22	38.49	60.00	27.99	22.22	38.49	60.07	43.47	44.56	38.59
Mental health	58.40	13.15	66.67	2.31	52.00	13.86	62.67	10.07	51.20	13.97	56.00	10.58

VAS: Visual Analog Scale; PSEQ: Pain Self-Efficacy Questionnaire; RMDQ: Roland Morris Disability Questionnaire; SF36: Short Form Health Survey; SM + PT: self-management + physiotherapy; PT: physiotherapy.

**Table 3:** Comparisons of group difference in VAS, PSEQ, and RMDQ over time.

Outcome measure	Within-group effect			Interaction effect	Between-group effect	
	Pre-mid	Mid-post	Pre-post		*F*	*p*
VAS	0.45	0.096	0.086	—	—	0.24
PSEQ	0.24	0.19	0.40	0.076	7.31	**0.035** ^*∗*^
Adjusted PSEQ	0.82	**0.033** ^*∗*^	0.56	**0.008** ^*∗*^	2.43	0.18
RMDQ	0.76	0.28	0.46	0.62	7.30	**0.035** ^*∗*^
Adjusted RMDQ	0.34	0.88	0.64	0.82	2.79	0.16

VAS: Visual Analog Scale; PSEQ: Pain Self-Efficacy Questionnaire; RMDQ: Roland Morris Disability Questionnaire; SM + PT: self-management + physiotherapy; PT: physiotherapy; pre-mid: pretreatment vs midterm; mid-post: midterm vs posttreatment; post-pre: posttreatment vs pretreatment; ^*∗*^*p* < 0.05.

**Table 4:** Comparisons of group difference in SF36 over time.

Outcome measure	Within-group effects			Interaction effect	Between-group effects	
	Pre-mid	Mid-post	Pre-post		*F*	*p*
Physical function	0.25	0.85	0.89	—	—	0.87
Role physical	0.41	0.68	0.16	—	—	0.48
Bodily pain	**0.023** ^*∗*^	0.10	**0.046** ^*∗*^	—	—	**0.008** ^*∗*^
General health	0.80	0.43	0.40	0.52	3.2	0.12
Adjusted general health	0.079	0.87	**0.033** ^*∗*^	0.069	2.12	0.21
Vitality	0.46	0.14	0.68	—	—	0.55
Social function	1.00	0.66	0.68	—	—	0.82
Role emotional	0.10	0.58	0.080	—	—	0.16
Mental health	0.079	0.58	**0.017** ^*∗*^	—	—	**0.013** ^*∗*^

SF36: Short Form Health Survey; SM + PT: self-management + physiotherapy; PT: physiotherapy; pre-mid: pretreatment vs midterm; mid-post: midterm vs posttreatment; post-pre: posttreatment vs pretreatment; ^*∗*^*p* < 0.05.
